# Prognostic value of PSMA PET/CT-Based local staging in predicting biochemical recurrence after radical prostatectomy

**DOI:** 10.1007/s00259-025-07455-0

**Published:** 2025-07-28

**Authors:** Vera Sweere, Alexandra Bruins Slot, Rick Hermsen, Joris G. Heetman, Lieke Wever, Jules Lavalaye, Maarten Vinken, Clinton D. Bahler, Mark Tann, Claudia Kesch, Tugce Telli, Wolfgang P. Fendler, Peter Ka-Fung Chiu, Fabio Zattoni, Laura Evangelista, Francesco Ceci, Marcin Miszczyk, Pawel Rajwa, Francesco Barletta, Alberto Briganti, Francesco Montorsi, Giorgio Gandaglia, Jean-Paul A. van Basten, Harm H.E. van Melick, Roderick C.N. van den Bergh, Giancarlo Marra, Matthijs J.V. Scheltema, Timo F.W. Soeterik

**Affiliations:** 1 Department of Urology, St. Antonius Hospital, Koekoekslaan 1, 3435 CM, Nieuwegein, Utrecht The Netherlands; 2 Department of Nuclear Medicine, Canisius Wilhelmina Hospital, Nijmegen, The Netherlands; 3 Department of Nuclear Medicine, St. Antonius Hospital, Nieuwegein, Utrecht The Netherlands; 4 Department of Urology, Indiana University Medical Center, Indianapolis, USA; 5 Department of Radiology and Imaging Sciences, Indiana University Medical Center, Indianapolis, USA; 6 Department of Urology, University Hospital Essen, Essen German Cancer Consortium (DKTK) University Hospital Essen, Essen, Germany; 7 Department of Nuclear Medicine, University Hospital Essen, Essen, Germany; 8 West German Cancer Center (WTZ), Essen, Germany; 9 German Cancer Consortium (DKTK), Essen, Germany; 10 S. H. Ho Urology Centre, Department of Surgery, The Chinese University of Hong Kong, Hong Kong, China; 11 Department of Surgery, Oncology, and Gastroenterology, Urological Unit, University of Padova, Padova, Italy; 12 Department of Biomedical Sciences, Humanitas University, Pieve Emanuele, Milan, Italy; 13 Division of Nuclear Medicine, IRCCS Humanitas Research Hospital, Milan, Italy; 14 Division of Nuclear Medicine and Theranostics, IEO European Institute of Oncology, IRCCS, Milan, Italy; 15 Department of Urology, Comprehensive Cancer Center, Medical University of Vienna, Vienna, Austria; 16 Collegium Medicum - Faculty of Medicine, WSB University, Dąbrowa Górnicza, Poland; 17 Second Department of Urology, Centre of Postgraduate Medical Education, Warsaw, Poland; 18 Division of Surgery & Interventional Science, University College London, London, UK; 19 Division of Oncology/Unit of Urology, Soldera Prostate Cancer Lab, URI, IRCCS San Raffaele Scientific Institute, Vita-Salute San Raffaele University, Milan, Italy; 20 Department of Urology, Canisius Wilhelmina Hospital, Nijmegen, The Netherlands; 21 Department of Urology, Erasmus Medical Center, Rotterdam, The Netherlands; 22 Department of Urology, University Hospital S Giovanni Battista, Azienda Ospedaliero Universitaria Città della Salute e della Scienza di Torino, Turin, Italy; 23 Department of Radiation Oncology, University Medical Center Utrecht, Utrecht, The Netherlands

**Keywords:** PSMA PET/CT, Prostate cancer, Biochemical recurrence-free survival, MRI

## Abstract

**Purpose:**

PSMA PET/CT outperforms conventional imaging for detecting pelvic nodal and distant metastasis, but its role regarding local staging and risk stratification remains unclear. This study aims to evaluate the association between PSMA PET/CT characteristics and biochemical recurrence-free survival (BRFS) after robot-assisted radical prostatectomy (RARP) in patients with prostate cancer.

**Methods:**

In this international multicentre retrospective study, we analyzed 476 patients with localized or locally advanced miN0 prostate cancer, staged with PSMA PET/CT and MRI before RARP (2016–2023). Predictors of BRFS were identified using univariate and multivariate Cox regression with backward elimination based on Akaike information criterion (AIC). Kaplan-Meier analysis assessed the association of clinical stage by MRI, PSMA PET, and their combination with BRFS.

**Results:**

In total 476 patients were included with a median follow-up of 18.0 months (IQR 6.9–29.3). Of the 127 BCRs, 101 (79.5%) occurred within two years post-surgery. The final multivariate model included initial PSA (10–20 vs. <10: HR 1.92 [95% CI 1.21–3.05]; >20 vs. <10: HR 2.26 [95% CI 1.30–3.93]), biopsy ISUP grade group (2–3 vs. 1: HR 2.28 [95% CI 0.70–7.41]; 4–5 vs. 1: HR 3.62 [95% CI 1.12–11.65]), MRI T-stage (T3a vs. ≤T2: HR 1.19 [95% CI 0.75–1.90]; ≥T3b vs. ≤T2: HR 2.09 [95% CI 1.21–3.62]), and PSMA PET T-stage (T3a vs. ≤T2: HR 1.05 [95% CI 0.59–1.85]; ≥T3b vs. ≤T2: HR 2.75 [95% CI 1.63–4.63]). From the full model, clinical T-stage, MRI-derived index diameter, SUV_max_, PSMA_total_ and PSMA_vol_ were eliminated. The 2-year BRFS was 19% (95% CI 6.7–51%) in patients with T3b disease on both MRI and PSMA PET compared to 58% (95% CI 40–84%) in those with T3b detected only on MRI (*p* = 0.03).

**Conclusion:**

Clinical tumor stage assessed by PSMA PET was independently associated with BRFS in multivariate analysis, adjusting for clinical parameters and MRI-derived staging. This suggests that incorporating PSMA PET-based local staging may improve risk stratification and guide treatment decisions.

**Supplementary Information:**

The online version contains supplementary material available at 10.1007/s00259-025-07455-0.

## Introduction

The European Association of Urology (EAU) risk classification, derived from the study by D’Amico et al. [1], categorizes patients into three risk groups (low, intermediate and high) according to their likelihood of biochemical recurrence (BCR); the high-risk group also encompasses patients with locally advanced disease. The National Comprehensive Cancer Network (NCCN) system divides each of these groups in two subgroups, yielding six risk groups for clinically localised prostate cancer (PCa) [2]. These strata guide both treatment selection and treatment intensity [3].

Approximately 20% of patients who undergo radical prostatectomy for localised PCa experience BCR within five years. Accurately identifying those at higher risk is therefore crucial for tailoring treatment intensity and potentially improving patient outcomes [4]. The current EAU risk classification system incorporates clinical stage based on digital rectal examination (DRE), serum PSA and biopsy International Society or Urological Pathology (ISUP) grade group (GG). However, prior studies have shown that magnetic resonance imaging (MRI) outperforms DRE in detecting extracapsular disease. Reliance on DRE alone can thus underestimate tumour extent and prognosis in a subset of patients [5]. To address this limitation, novel risk classification systems incorporating MRI data have been developed, potentially enhancing the accuracy of initial disease prognosis and guiding more precise treatment selection [6, 7].

Prostate-specific membrane antigen (PSMA) positron emission tomography/computed tomography (PET/CT) has proven to be a suitable replacement for conventional imaging with superior accuracy to the combined findings of CT and bone scanning [8]. When combined with MRI, PSMA PET/CT further improves local staging by enhancing the detection of extraprostatic extension and seminal vesicle invasion [9, 10]. Mookerji et al., showed that PSMA PET/CT alone outperformed MRI in accurately identifying the tumour stage [11]. Besides its value in prostate cancer staging, PSMA PET/CT enables the quantification of imaging parameters, such as the maximum standardized uptake value (SUV_max_), which represents the highest uptake value within a single voxel of the region of interest. PSMA parameters have been shown to be associated with pathologic ISUP grade group and lymph node involvement [12–14]. In a number of smaller single centre studies, these parameters were also shown to be associated with BCR, further emphasizing their potential to enhance risk stratification [15–17]. PSMA PET-based risk systems were found to better stratify patients with regard to overall survival than established clinical tools [18].Given the potential of PSMA PET to further improve disease risk stratification, large multicentre studies are needed to evaluate the association of PSMA PET local staging information and quantitative parameters with longer-term outcomes such as biochemical recurrence free survival (BRFS).

The aim of this international multicentre study is to evaluate the association between PSMA PET/CT-derived local staging and quantitative parameters (i.e., SUV_max_, PSMA_vol_, PSMA_total_) with BRFS in miN0 patients after radical prostatectomy. Additionally, the prognostic significance of PSMA PET-detected extra prostatic extension or seminal vesicle invasion compared to localised disease on MRI remains unclear. By analysing these associations in a contemporary patient cohort, we aim to clarify PSMA PET/CT’s predictive utility for BRFS and to inform more precise risk-adapted treatment strategies.

## Methods

### Study cohort

We retrospectively collected data from seven national and international tertiary referral centres on 579 men with histopathologically confirmed, clinically localised prostate cancer who underwent radical prostatectomy, with or without pelvic lymph node dissection. Patients were included if they had undergone both preoperative PSMA PET/CT and MRI between 2016 and 2023 and had a minimum of 6 months follow-up. Patients were excluded if they had prior focal and/or systemic treatment for PCa; if lymph node metastases were detected on PSMA PET/CT (*n* = 70); or if PSMA PET/CT was used as the sole pre-operative staging modality, i.e., without MRI (*n* = 33). Written informed consent was waived due to the retrospective nature of the study. The Medical research Ethics Committees United (MEC-U) has reviewed the study protocol, and waived the need for (MEC-U) approval (AW24.048/W18.055). Institutional Review Boards approved of the study prior to initiating data collection.

### Data collection

Preoperative parameters that were collected included age, initial prostate specific antigen (iPSA), clinical tumor stage assessed by DRE, EAU risk classification based upon d’Amico risk classification, MRI T-stage, highest Prostate Imaging-Reporting and Data System (PI-RADS) score, and biopsy International Society of Urological Pathology (ISUP) grade group. Furthermore, PSMA T-stage and PSMA parameters, including PSMA_vol_, SUV_max_ and PSMA_total_, and types of radiotracers were collected. The outcomes were BCR and BRFS. BCR was defined as either two consecutive PSA measurements of ≥ 0.2 ng/ml or a single elevated PSA measurement followed by salvage therapy. BRFS was defined as the time between surgery and BCR or last follow-up.

### MRI acquisition and interpretation

MRI scans were evaluated by experienced uro-radiologists following PI-RADS version 2.1 criteria. Both multiparametric MRI (mpMRI: T1W, T2W, DWI, and DCE sequences) and biparametric MRI (bpMRI: T2W and DWI, excluding DCE) were accepted.

### PSMA PET/CT procedures

All PSMA PET/CT scans were performed at tertiary centres according to local protocols. External scans were accepted and were re-evaluated by the local team. PET/CT images were acquired from the mid-thigh to the skull base and combined with either low-dose or diagnostic CT for anatomical correlation. All scans were reviewed by an experienced nuclear medicine physician (> 5 years of experience and/or > 500 studies) or trained research fellow under the direct supervision of a nuclear medicine staff physician, both blinded to MRI results. The radiotracers that were used included [^68^Ga]Ga-PSMA-11, [^18^F]PSMA-1007, [^18^F]DCF-PyL, and [^18^F]-JK-PSMA-7, with acquisition performed according to European Association of Nuclear Medicine/Society of Nuclear Medicine and Molecular Imaging guidelines [13]. Local staging was reassessed using the PROMISE v2 miT classification [19]. PSMA PET/CT procedures were performed as described previously [14].

### PSMA PET/CT parameters

Quantitative PSMA parameters were assessed as described previously [14]. The quantitative PSMA parameters evaluated included SUV_max_, (which is the highest uptake value within a single voxel of the region of interest, g/mL), PSMA-positive volume (PSMA_vol_, mL) (which was determined using an absolute cut-off at SUV ≥ 4) and total PSMA accumulation (PSMA_total_,, SUV · mL), defined as PSMA_vol_ × SUV_mean_ of the volume of interest [20].

### Statistical analysis

Before conducting the analyses, iPSA, clinical stage assessed by DRE and biopsy ISUP grade group were stratified in accordance with the EAU risk classification (e.g. PSA 0–10, 10–20 and > 20; T1c-T2a, T2b and T2c-T4; biopsy ISUP GG 1, 2–3 and 4–5 subgroups) [3]. MRI T-stage and MRI-derived diameter of index lesion (IL) were categorized in accordance with Mazzone et al. (T2, T3a and T3b-T4; diameter IL < 10, 10–20, >20 mm) [7]. PSMA T-stage was subdivided as follows: T1c-T2, T3a and T3b-T4. Univariate Cox regression analysis was performed to evaluate the associations between clinical parameters, MRI characteristics, PSMA PET/CT clinical staging and quantitative parameters, and BRFS.

The multivariate cox-regression model was estimated using backward stepwise approach minimizing the Akaike information criterion (AIC) [21, 22]. The initial model included variables, which have previously been shown to be associated with prostate cancer outcomes: iPSA; clinical T-stage; biopsy ISUP; MRI T-stage; MRI-derived diameter of index lesion; PSMA PET T-stage; PSMA PET quantified parameters (SUV_max_, PSMA_total_ and PSMA_vol_) [3, 7]. Next, we conducted a collinearity analysis of the variables included in the final model using the Variance Inflation Factor (VIF). A VIF between 1 and 5 indicates low to moderate collinearity, while a VIF greater than 5 suggests high collinearity [23].

To assess the influence of PSMA ligand type on model performance, we conducted a sub-analysis stratified by the most frequently used radiotracers, [¹⁸F]PSMA-1007 and [⁶⁸Ga]Ga-PSMA-11, and recalculated the concordance index within each cohort.

To evaluate the prognostic value of extra prostatic extension and seminal vesicle involvement on PSMA PET/CT, both independently and in comparison to MRI, Kaplan-Meier curves and log-rank tests were conducted. All statistical analyses were performed using R version 4.4.3. All tests were two-sided, with a significance threshold set at *p* < 0.05.

## Results

### Baseline characteristics

A total of 476 patients were eligible. The median age at surgery was 66 years (interquartile range [IQR] 62.0–71.0]), and the median iPSA was 9.6 ng/mL [IQR 6.4–16.0]. Median iPSA was higher in patients who developed BCR (12.9 ng/mL [IQR 8.7–22.0]).

Overall, 2.1%, 44.3%, and 53.6% of patients were classified as EAU low-, intermediate-, and high-risk prostate cancer. MRI staging most commonly showed T2a (*n* = 189, 39.7%) or T3a (*n* = 155, 32.6%) disease. According PSMA PET/CT clinical staging, 338 (71.0%), 84 (17.6%), and 52 (11.4%) patients had T1c–T2, T3a, and T3b–T4 disease, respectively. The median SUV_max_, PSMA_vol_, and PSMA_total_ were 9.5 g/mL [IQR 6.0–15.6], 4.5 mL [IQR 1.2–9.9], and 27.4 SUV·mL [IQR 6.3–70.5], respectively. [^68^Ga]Ga-PSMA-11 was the most frequently used radiotracer (*n* = 283, 59.5%), followed by [^18^F]PSMA-1007 (*n* = 171, 35.9%) (Table 1). BCR occurred in 84 (29.6%) patients of the [^68^Ga]Ga-PSMA-11 cohort and 37 (21.6%) of the [^18^F]PSMA-1007 cohort.

**Table 1:** Clinical characteristics and imaging-derived data, including MRI and PSMA PET/CT

	All patients (*n* = 476)
Age (years), median (IQR)	66.0 (62.0–71.0)
PSA (ng/ml), median (IQR)	9.6 (6.4–16.0)
Clinical T-stage, n (%)	
T1c	234 (49.2)
T2a	137 (28.8)
T2b	33 (6.9)
T2c	8 (1.7)
T3a	51 (10.7)
T3b	8 (1.7)
Missing	5 (1.1)
Biopsy ISUP, n (%)	
1	31 (6.5)
2	128 (26.9)
3	134 (28.2)
4	122 (25.6)
5	61 (12.8)
EAU risk classification, n (%)	
Low	10 (2.1)
Intermediate	211 (44.3)
High	255 (53.6)
MRI findings, n (%)	
MRI T-stage	
T1c	14 (2.9)
T2a	189 (39.7)
T2b	20 (4.2)
T2c	53 (11.1)
T3a	155 (32.6)
T3b	44 (9.2)
T4	1 (0.2)
PI-RADS	
1	14 (2.9)
2	4 (0.8)
3	40 (8.4)
4	138 (29.0)
5	243 (51.1)
Missing	37 (7.8)
MRI-derived size index lesion (mm)	
< 10	109 (22.9)
10–20	158 (33.2)
> 20	176 (37.0)
Missing	32 (12.8)
PSMA PET/CT findings, n (%)	
PSMA T-stage	
T1c	21 (4.4)
T2	317 (66.6)
T3a	84 (17.6)
T3b	48 (10.1)
T4	6 (1.3)
PSMA_vol_ (cm^3^), median (IQR)	4.5 (1.2–9.9)
PSMA_total_, median (IQR)	27.4 (6.3–70.5)
SUV_max_, median (IQR)	9.5 (6.0-15.6)
Tracer	
68Ga-PSMA-11	283 (59.5)
18 F-PSMA-1007	171 (35.9)
18 F-DCFPyL	5 (1.1)
18 F-JK-PSMA-7	17 (3.6)

*PSA* prostate specific antigen, *MRI* magnetic resonance imaging, *PI-RADS* Prostate Imaging-Reporting and Data System, *ISUP* International Society or Urological Pathology, *PSMA* prostate specific membrane antigen, *EAU *European Association of Urology

Surgical histopathological outcomes are presented in Table 2. Histopathological lymph node involvement was confirmed in 50 patients (10.5%). Among these, BCR occurred in 24/50 (48.0%).

**Table 2:** Final histopathological results of radical prostatectomy specimen

	All patients (*n* = 476)	BCR (*n* = 127)	No-BCR (*n* = 349)
Pathological T-stage, n (%)			
T2	38 (24.4)	9 (7.1)	29 (8.3)
T2a	5 (4.5)	1 (0.8)	4 (1.1)
T2c	40 (27.6)	7 (5.5)	33 (9.5)
T3a	35 (25.0)	17 (13.4)	18 (5.2)
T3b	27 (18.6)	17 (13.4)	10 (2.9)
Missing	331 (69.5)	76 (59.8)	255 (73.1)
Pathological N-stage, n (%)			
N0	348 (73.1)	90 (70.9)	258 (73.9)
N1	50 (10.5)	26 (20.5)	24 (6.9)
Nx	75 (15.8)	9 (7.1)	66 (18.9)
Missing	3 (0.6)	2 (1.6)	66 (18.9)
Positive margins, n (%)			
No	250 (52.5)	54 (42.5)	196 (56.2)
Yes	98 (20.5)	46 (36.2)	52 (14.9)
Missing	128 (26.9)	27 (21.3)	101 (28.9)
Surgical ISUP, n (%)			
1	10 (2.1)	0 (0.0)	10 (2.9)
2	191 (40.1)	29 (22.8)	162 (46.4)
3	175 (36.8)	54 (42.5)	121 (34.7)
4	49 (10.3)	22 (17.3)	27 (7.7)
5	47 (9.9)	19 (15.0)	28 (8.0)
Missing	4 (0.8)	3 (2.4)	1 (0.3)

*pT* pathologic T-stage, *pN* pathologic N-stage, *ISUP* International Society or Urological Pathology

### Biochemical recurrence-free survival

The median follow-up for all patients was 18.0 months (IQR 6.9–29.3). In total, 127 patients experienced BCR, which occurred in 60 (47.2%) cases within one year and in 101 (79.5%) cases within two years post-surgery. The median follow-up for patients without BCR was 20.4 months (IQR 8.1–32.2).

### Univariate Cox regression analysis

In univariate Cox regression analysis, PSMA PET/CT-derived T3b stage was significantly associated with BCR (HR 3.809, 95% CI 2.514–5.772; *p* < 0.001). Additionally, SUV_max_, PSMA_vol_, and PSMA_total_ were also significantly associated with BCR, each demonstrating an increased hazard: SUV_max_ (HR 1.025, 95% CI 1.009–1.040), PSMA_vol_ (HR 1.014, 95% CI 1.006–1.023), and PSMA_total_ (HR 1.002, 95% CI 1.001–1.003) (all *p* < 0.001). A summary of all univariate Cox regression analyses is provided in Table 3.

**Table 3:** Univariate cox-regression analyses to assess the individual correlations between PSMA PET/CT-parameters and BRFS

Variables					
		HR	95%CI		*P* value
PSA level (ng/ml)	< 10	Ref			
	10–20	2.265	1.485	3.454	**< 0.001**
	> 20	3.133	1.985	4.953	**< 0.001**
Clinical T-stage	≤T2a	Ref			
	T2b	1.820	1.191	2.780	**0.006**
	≥T2c	2.120	1.324	3.396	**0.002**
MRI-based imaging stage	≤T2	Ref			
	T3a	1.389	0.940	2.052	0.099
	≥T3b	2.834	1.762	4.558	**< 0.001**
MRI-derived index lesion diameter (mm)	< 10	Ref			
	10–20	1.721	0.932	3.178	0.083
	> 20	2.422	1.334	4.397	**0.004**
Biopsy ISUP grade	1	Ref			
	2–3	1.784	0.644	4.943	0.265
	4–5	3.202	1.169	8.770	**0.024**
PSMA-based imaging stage	T2	Ref			
	T3a	1.040	0.615	1.759	0.884
	T3b-T4	3.809	2.514	5.772	**< 0.001**
SUV_max_		1.025	1.009	1.040	**< 0.001**
PSMA_vol_		1.014	1.006	1.023	**< 0.001**
PSMA_total_		1.002	1.001	1.003	**< 0.001**

*CI* confidence interval, *HR* hazard ratio, *PSMA* prostate specific membrane antigen, *SUV*_max_ maximum standardized uptake value, *PSMA*_vol _PSMA_volume_

### Multivariate Cox regression analysis

The multivariate Cox regression model, incorporating all potential predictors, including iPSA, clinical T-stage, MRI T-stage, MRI-derived index lesion diameter, biopsy ISUP grade group, PSMA PET/CT T-stage, SUV_max_, PSMA_total_, and PSMA_vol_, demonstrated a concordance index of 0.731 (95% CI 0.679–0.783).

Following backward elimination (Supplementary Table 1), the final model retained iPSA (10–20 vs. <10: HR 1.919 [95% CI 1.208–3.049]; >20 vs. <10: HR 2.264 [95% CI 1.304–3.931]), biopsy ISUP grade (2–3 vs. 1: HR 2.275 [95% CI 0.699–7.406]; 4–5 vs. 1: HR 3.618 [95% CI 1.124–11.648]), MRI T-stage (T3a vs. ≤T2: HR 1.190 [95% CI 0.746–1.899]; ≥T3b vs. ≤T2: HR 2.092 [95% CI 1.210–3.618]), and PSMA PET/CT T-stage (T3a vs. ≤T2: HR 1.048 [95% CI 0.593–1.854]; ≥T3b vs. ≤T2: HR 2.746 [95% CI 1.628–4.632]) (Table 4).

**Table 4:** Refined multivariate cox-regression model after backward elimination integrating radiological and clinical parameters predicting BCR after radical prostatectomy

Variables		Multivariate model			
		HR	95%CI		*P* value
PSA level (ng/ml)	< 10	Ref			
	10–20	1.919	1.208	3.049	**0.006**
	> 20	2.264	1.304	3.931	**0.004**
MRI-based imaging stage	≤T2	Ref			
	T3a	1.190	0.746	1.899	0.466
	≥T3b	2.092	1.210	3.618	**0.008**
Biopsy ISUP grade	1	Ref			
	2–3	2.275	0.699	7.406	0.172
	4–5	3.618	1.124	11.648	**0.031**
PSMA-based imaging stage	T2	Ref			
	T3a	1.048	0.593	1.854	0.871
	T3b-T4	2.746	1.628	4.632	**< 0.001**

*CI* confidence interval, *HR* hazard ratio, *PSA* prostate specific antigen, *MRI* magnetic resonance imaging, *ISUP* International Society of Urological Pathology, *PSMA* prostate specific membrane antigen

The final model demonstrated a lower AIC (981.2 vs. 991.8) and a concordance index of 0.722 (95% CI 0.680–0.783). Stepwise variable removal based on minimization of the AIC resulted in only a slight change in the concordance index (0.731 [95% CI 0.679–0.783 vs. 0.722 [95% CI 0.680–0.783]), yielding the most parsimonious model while preserving discriminatory performance. Collinearity analysis showed a weak-to-moderate collinearity between all parameters (Supplementary Table 2). In our sub-analysis, stratification by radiotracer yielded concordance indices of 0.67 (95% CI 0.55–0.78) for [¹⁸F]PSMA-1007 and 0.77 (95% CI 0.69–0.86) for [⁶⁸Ga]Ga-PSMA-11.

### Prognostic impact of stage ≥ T3b on MRI and PSMA PET/CT

**Fig. 1:**
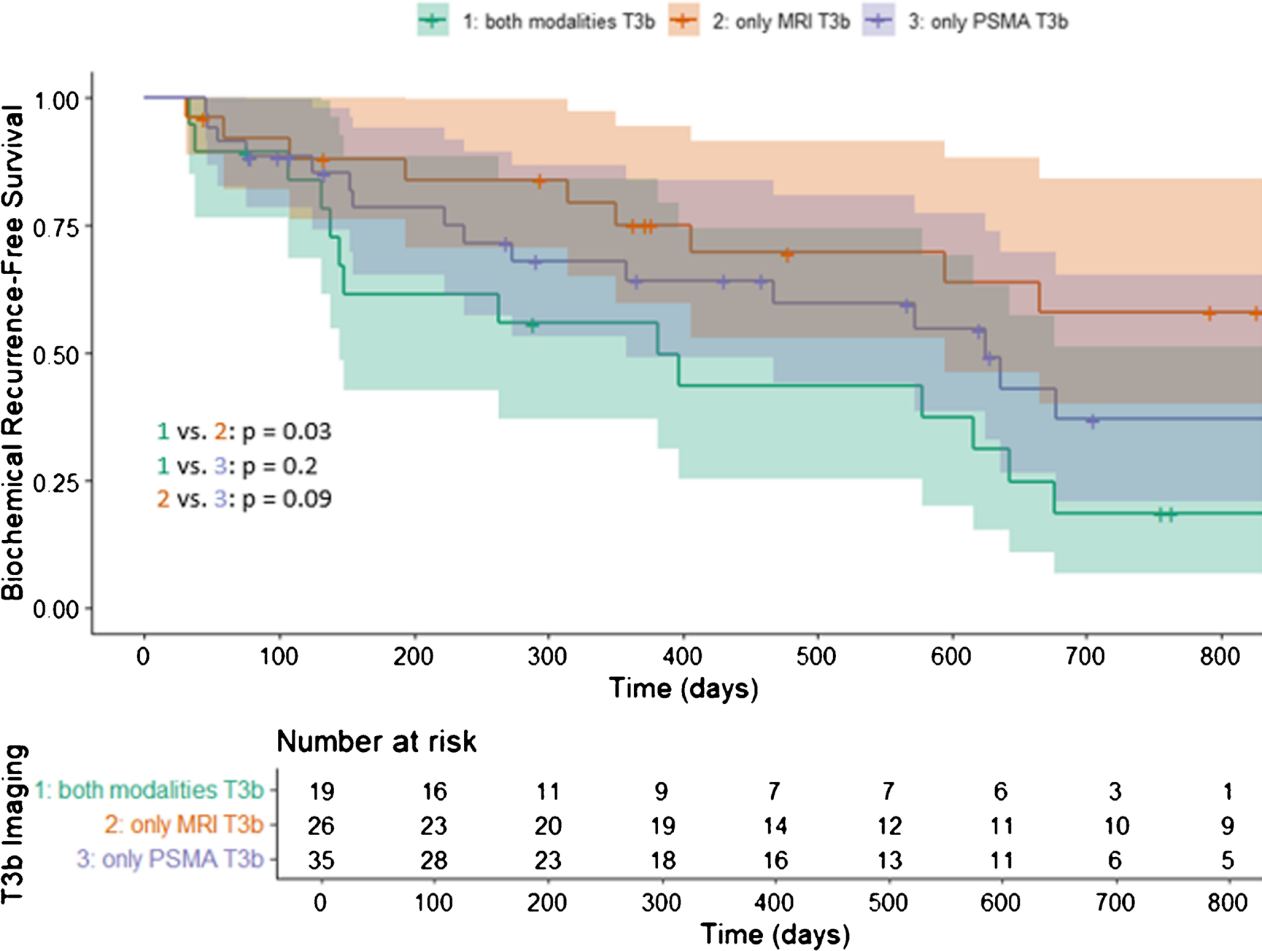
Kaplan Meier curves assessing biochemical progression-free survival of all included patients who underwent a radical prostatectomy, stratified by: 1) ≥ T3b on PSMA and MRI; 2) only MRI ≥ T3b; 3) only PSMA ≥ T3b

The 2-year BRFS rate was 18.6% [95% CI 6.7–51.2%] in patients with stage ≥ T3b on both MRI and PSMA PET/CT, compared to 58.1% [95% CI 40.1–84.3%] in those with stage ≥ T3b detected only on MRI (log-rank *p* = 0.03). In patients with stage ≥ T3b detected only on PSMA PET/CT, the 2-year BRFS rate was 36.9% [95% CI 20.9–65.2%] (Fig. 1).

## Discussion

This study examined whether PSMA PET/CT-derived tumour stage and quantitative parameters improve prediction of BRFS in patients without suspected lymph node involvement on molecular imaging. Our multivariate analysis identified PSMA PET/CT local staging as a significant predictor of BCR risk. Additional variables retained in the final model included initial PSA, biopsy ISUP grade, and MRI T-stage. Importantly, patients with PCa classified as T3b on both PSMA PET/CT and MRI had a significantly greater risk of recurrence than those in whom T3b disease was detected by MRI alone. These findings demonstrate that PSMA PET/CT provides prognostic information beyond conventional clinical factors and should be incorporated into initial staging to refine risk stratification and guide treatment intensity.

Our findings are concordant with prior studies evaluating the prognostic relevance PSMA PET staging of primary prostate cancer. Meijer et al. found that higher initial PSA value, biopsy GG ≥ 4, ≥T3 disease on mpMRI and miN1 disease on PSMA PET/CT were predictors for BCR after radical prostatectomy [24]. In addition, Baas et al. showed that the suspicion of lymph node invasion in PSMA PET/CT was a significant predictor of BCR [25]. More recently, Karpinski et al. assessed the prognostic value of PSMA PET by PROMISE (PPP) nomograms, showing that these models achieve comparable or superior predictive accuracy to established risk classification systems such as EAU, NCCN, STAR-CAP, and GAFITA [2, 3, 26, 27].

To our knowledge, this is the first large multicentre study demonstrating the independent prognostic value of PSMA PET/CT local staging in addition to MRI-derived staging in patients with localised or locally advanced miN0 prostate cancer. These findings highlight the potential of PSMA PET/CT to refine risk stratification beyond nodal assessment. Future research should investigate whether integrating PSMA PET/CT with MRI staging enhances prediction of long-term outcomes, including distant metastasis-free and overall survival [28].

A key strength of this study is the evaluation of the prognostic value of quantitative PSMA PET parameters (i.e., SUV_max_, PSMA_vol_, PSMA_total_). In univariate analysis, these parameters were significantly associated with BCR, consistent with previous studies regarding the predictive value of PSMA PET/CT [15–17]. However, in multivariate analysis, quantitative PSMA PET parameters did not significantly improve model performance when added to PSMA PET/CT local staging, MRI T-stage, biopsy ISUP grade, and iPSA. These findings are inconsistent with those reported by Roberts et al., who reported that incorporating SUV_max_ into a multivariate model, including age, iPSA, biopsy Gleason score, and PI-RADS, enhanced BRFS prediction [29]. The divergence from the results of Roberts et al. is likely driven by differences in model composition. Roberts’ model did not include PSMA PET/CT-derived T stage, nor did it account for MRI T stage, both of which we incorporated. In our cohort, PSMA PET/CT T stage proved to be an independent and more powerful predictor of BRFS than SUV_max_; its strong and overlapping association with BRFS led AIC-based selection to drop SUV_max_ from the final model.

Our study provides novel insights by demonstrating that PSMA PET/CT-derived clinical stage has superior prognostic value compared to quantitative PET parameters and remains an independent prognostic factor after adjustment for MRI T-stage and clinical covariates. These findings suggest that while quantitative PSMA PET parameters may offer additional biological insights, they cannot replace the well-established TNM staging system. Clinically, PSMA PET/CT-derived clinical staging may also offer advantages due to its standardized nature and strong inter-observer reproducibility [30]. In contrast, uptake-based metrics such as SUV_max_ are subject to variability and can be influenced by non-oncological factors, such as type of radiotracer [31].

A theoretical advantage of [¹⁸F]PSMA-1007 over [⁶⁸Ga]Ga-PSMA-11 is its minimal urinary clearance, potentially improving lesion detection near the prostate bed [32]. However, this presumed benefit was not confirmed in earlier analyses, which reported lower sensitivity for [¹⁸F]PSMA-1007 (33% vs. 48%) but slightly higher specificity (88% vs. 80%) compared to [⁶⁸Ga]Ga-PSMA-11 [9]. Direct comparisons of their ability to predict BCR remain scarce. In our sub-analysis, concordance indices were 0.67 (95% CI 0.55–0.78) for [¹⁸F]PSMA-1007 and 0.77 (95% CI 0.69–0.86) for [⁶⁸Ga]Ga-PSMA-11. The lower discriminative ability may reflect slightly inferior staging performance. Caution is warranted, as 90.1% of [¹⁸F]PSMA-1007 cases originated from a single centre, suggesting possible centre-related bias. Furthermore, this group included fewer patients (*n* = 171) and BCR events (*n* = 37), potentially limiting model robustness. To conclude, clarifying the prognostic implications of tracer selection will require harmonised analyses across larger and more diverse clinical cohorts.

Beyond radiotracer-related factors, advances in scanner technology may also shape diagnostic and prognostic performance. PSMA PET/MRI combines molecular imaging with the superior soft tissue contrast of MRI. This is supported by a study from Muehlematter et al., which found higher sensitivity for detecting extraprostatic extension compared to mpMRI [33]. Evidence on the prognostic value of PSMA PET/MRI remains limited. However, a meta-analysis by Evangelista et al. demonstrated that PSMA PET/MRI outperforms PET/CT in the detection of prostate cancer lesions, both in primary staging and in restaging during biochemical recurrence [34]. Also, Moradi et al. showed SUV_max_ >12.5 on PSMA PET/MRI to be significantly associated with biochemical failure and rapid recurrence after radical prostatectomy [35]. Further prospective studies are warranted to clarify the prognostic role of PSMA PET/MRI.

Our study has several limitations. First, the relatively short follow-up duration (median 18.0 months, IQR 6.9–29.3) may limit long-term outcome assessment. Nevertheless, the number of BCR events per variable was sufficient for statistical modelling and robust analysis. Second, the number of patients with T3b disease on PSMA PET/CT and MRI was relatively low, which may limit statistical power for subgroup analyses. Third, the lack of centralized review for MRI, PSMA PET/CT, and histopathology may have introduced variability in image interpretation and pathological assessment. Fourth, men who received both MRI and PSMA constitute a selection of cases. Lastly, imaging protocols varied across participating institutions, which may limit the generalizability of the results. Although MRI examinations were conducted at experienced centres and interpreted using the PI-RADS v2.1 criteria, acquisition protocols were not standardized across sites, and detailed technical parameters were not consistently reported. This lack of uniformity may have introduced inter-centre variability. However, this can also be regarded as a strength, as the heterogeneity represents the real-world clinical situation.

## Conclusion

PSMA PET/CT-derived T-stage, particularly stage ≥ T3b, is an independent predictor of BRFS in patients with localised or locally advanced miN0 PCa. We recommend integrating PSMA PET/CT T-stage into clinical decision-making alongside PSA, biopsy ISUP grade group, and MRI T-stage. While quantitative PSMA PET parameters such as SUV_max_ are significantly associated with BRFS, their predictive value diminishes when adjusted for these established risk factors. These results highlight the added prognostic value of PSMA PET/CT local staging and its potential role in guiding personalized treatment strategies.

## Supplementary information

Below is the link to the electronic supplementary material.

Supplementary file1 (DOCX 157 KB)

## Data Availability

The datasets generated during and/or analyzed during the current study are available from the corresponding author on reasonable request.
