# Supplementary material for: Prognostic value of PSMA PET/CT-Based local staging in predicting biochemical recurrence after radical prostatectomy
Source: Eur J Nucl Med Mol Imaging. 2025 Jul 28;53(2):921–30. doi: 10.1007/s00259-025-07455-0 (PMC12830398; doi:10.1007/s00259-025-07455-0)
Supplement: Supplementary file 1 — Supplementary file1 (DOCX 157 KB) [file 259_2025_7455_MOESM1_ESM.docx]

**Supplementary files**

**Supplementary table 1: Backward elimination by minimizing the Akaike Information Criterion (AIC)**


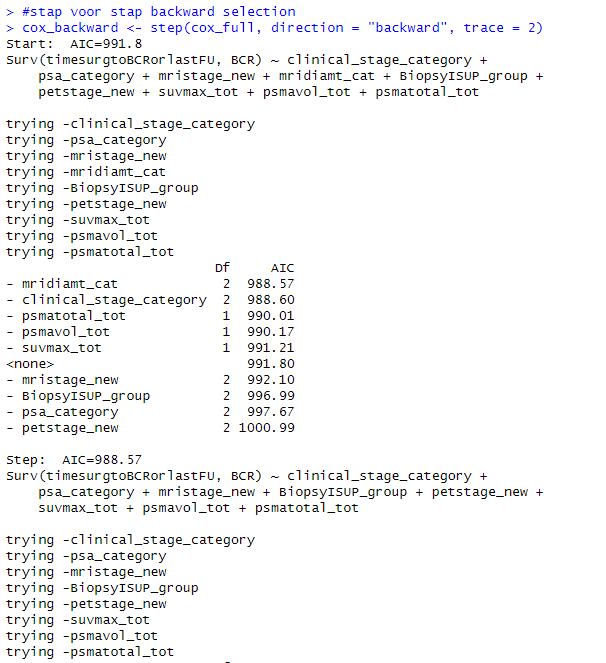


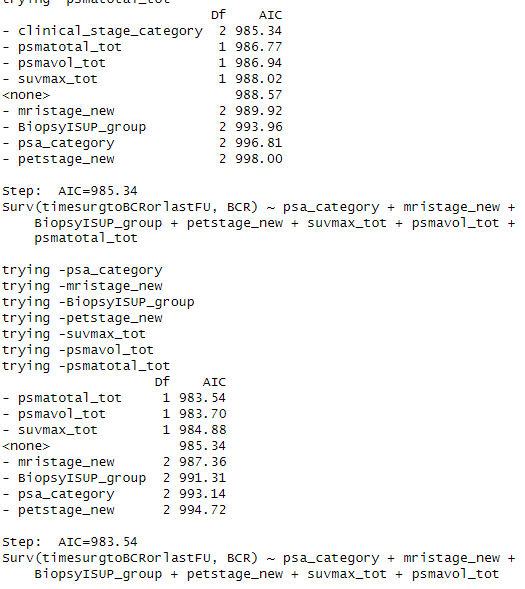


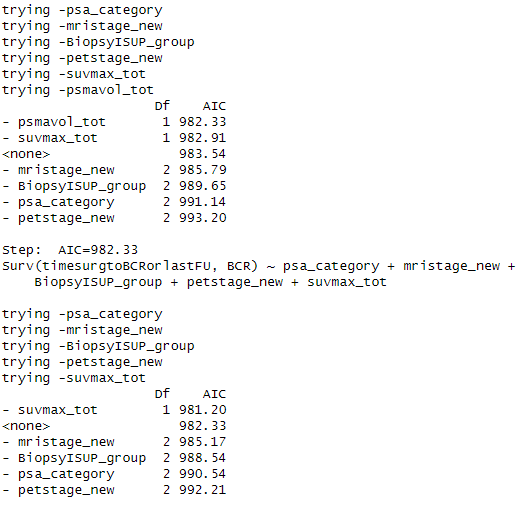


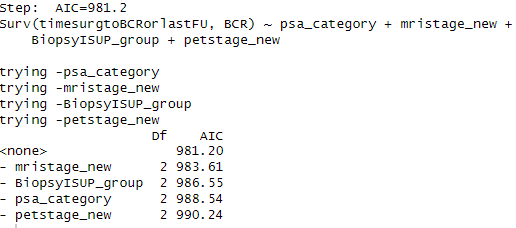


**Supplementary table 2: Multicollinearity of variables in backward cox-regression model evaluated by using Variance Inflation Factor (VIF)**

| Variable | VIF |
| --- | --- |
| PSA | 1.09 |
| MRI T-stage | 1.17 |
| Biopsy ISUP | 1.04 |
| PSMA T-stage | 1.24 |
